# Integrative Approach with Electrophysiological and Theoretical Methods Reveals a New Role of S4 Positively Charged Residues in PKD2L1 Channel Voltage-Sensing

**DOI:** 10.1038/s41598-017-10357-3

**Published:** 2017-08-29

**Authors:** Tomohiro Numata, Kunichika Tsumoto, Kazunori Yamada, Tatsuki Kurokawa, Shinichi Hirose, Hideki Nomura, Mitsuhiro Kawano, Yoshihisa Kurachi, Ryuji Inoue, Yasuo Mori

**Affiliations:** 1 Department of Physiology, Graduate School of Medical Sciences, Fukuoka University, 7-45-1 Nanakuma, Johnan-ku, Fukuoka, Fukuoka, 814-0180 Japan; 2 Laboratory of Molecular Biology, Department of Synthetic Chemistry and Biological Chemistry, Graduate School of Engineering, Kyoto University, Katsura, Nishikyo-ku, Kyoto, Kyoto, 615-8510 Japan; 3 Department of Pharmacology, Graduate School of Medicine, Osaka University, 2-2 Yamada-oka, Suita, Osaka 565-0871 Japan; 4 Division of Rheumatology, Department of Internal Medicine, Kanazawa University Graduate School of Medicine, 13-1 Takara-machi, Kanazawa, Ishikawa, 920-8641 Japan; 5 Department of Advanced Research in Community Medicine, Kanazawa University Graduate School of Medical Sciences, 13-1 Takara-machi, Kanazawa, Ishikawa, 920-8641 Japan; 6 Department of Pediatrics School of Medicine, Fukuoka University, 7-45-1 Nanakuma, Johnan-ku, Fukuoka, Fukuoka, 814-0180 Japan; 7 Department of General Medicine, Kanazawa University Hospital, 13-1 Takara-machi, Kanazawa, Ishikawa, 920-8641 Japan

## Abstract

Numerical model-based simulations provide important insights into ion channel gating when experimental limitations exist. Here, a novel strategy combining numerical simulations with patch clamp experiments was used to investigate the net positive charges in the putative transmembrane segment 4 (S4) of the atypical, positively-shifted voltage-dependence of polycystic kidney disease 2-like 1 (PKD2L1) channel. Charge-neutralising mutations (K452Q, K455Q and K461Q) in S4 reduced gating charges, positively shifted the Boltzmann-type activation curve [i.e., open probability (*P*_open_)-*V* curve] and altered the time-courses of activation/deactivation of PKD2L1, indicating that this region constitutes part of a voltage sensor. Numerical reconstruction of wild-type (WT) and mutant PKD2L1-mediated currents necessitated, besides their voltage-dependent gating parameters, a scaling factor that describes the voltage-dependence of maximal conductance, *G*_max_. Subsequent single-channel conductance (*γ*) measurements revealed that voltage-dependence of *G*_max_ in WT can be explained by the inward-rectifying property of *γ*, which is greatly changed in PKD2L1 mutants. Homology modelling based on PKD2 and Na_V_Ab structures suggest that such voltage dependence of *P*_open_ and *γ* in PKD2L1 could both reflect the charged state of the S4 domain. The present conjunctive experimental and theoretical approaches provide a framework to explore the undetermined mechanism(s) regulating TRP channels that possess non-classical voltage-dependent properties.

## Introduction

The integration of computer simulations with experimental biology has paved the way for new interdisciplinary science and technology that enables an understanding of systems biology. This new methodological approach has been extended to explore the fusion between theoretical equations and biological systems^1, 2^. Representative examples involve the modelling of excitable cells and systems in the squid giant axon^3^ and the use of a model of cardiac electrical activity^4^.

Transient receptor potential (TRP) channels are activated by a broad spectrum of physical and chemical stimuli generated from external sources and the interior of cells, and contribute to a variety of biological functions and the pathogenesis of many diseased states^5, 6^. Although TRP channels are generally considered to be non-voltage-gated, activation of several TRP channels show intrinsic voltage-dependency^7^, including TRPV1^8–10^, TRPV3^11^, TRPV5^12^, TRPV6^13^, TRPM3^14^, TRPM4^15^, TRPM5^16^, TRPM8^17^, TRPC5^18^, PKD2L1^19^ and TRPA1^20^. However, the mechanism(s) describing how these channels exert voltage-sensing and gating remains largely unknown.

The key structural components of classical voltage-gated ion channels are the voltage-sensing (S1–S4) and pore (S5 and S6) domains, which are linked by a short S4–S5 linker. The pore domain serves as an ion conduction pathway with a well-defined selectivity filter for specific ions. Importantly, S4 has a cluster of positively charged amino acids (AA) spaced every three residues, which are highly conserved and thought to play a key role in converting the change to the transmembrane potential to the conformational change of the pore domain that causes open-close transitions of the channels^21, 22^. AA sequence alignment between PKD2L1 channels and classical voltage-gated channels, i.e. voltage gated calcium (Ca_V_) channels, voltage-gated potassium (K_V_) channels and voltage gated sodium (Na_V_) channels, reveals that PKD2L1 has a few AA residues reminiscent of a voltage-sensor^23^. Indeed, recent structural analyses of K_V_1.2 and PKD2 suggest that the S4–S5 linker interacts with the C-terminus of S6, and mechanical movement of this linker leads to constriction or dilatation of the channel pore^24, 25^.

One common approach to investigate the voltage dependency is to construct the chord conductance-voltage relationship (*G*-*V* curve), which is expressed in the form of the Boltzmann equation. However, voltage-dependency of PKD2L1 causes a time-dependent shift and evaluation of its maximal activation, which only occurs at extremely positive potentials and shows varying extents of inactivation, is technically difficult to perform^19^.

In the present study, we undertake a complementary approach combining mutagenesis experiments and mathematical model-based simulations to more precisely understand the molecular mechanism underlying the voltage dependency of the PKD2L1 channel. To this end, we first characterised both wild-type (WT) and mutant PKD2L1 channels to compare their differences in voltage-dependent sensing/gating. The results indicated that the voltage dependence of these channels might be attributable to a similar voltage-sensing mechanism involved in classical voltage gated channels. We then attempted to faithfully construct a numerical model that reproduces these channel-gating properties based on experimental data obtained by voltage jump experiments. However, this modelling required an additional scaling factor to adjust the maximal conductance, which depends on the polarity of the membrane potential. The biophysical significance of this factor was further sought in terms of single channel recording. Finally, a possible structural basis for the observed voltage-dependence of PKD2L1 was explored by homology modelling based on the atomic structures of PKD2 and Na_V_Ab.

## Result

### The PKD2L1 channel has voltage dependency

**Figure 1:**
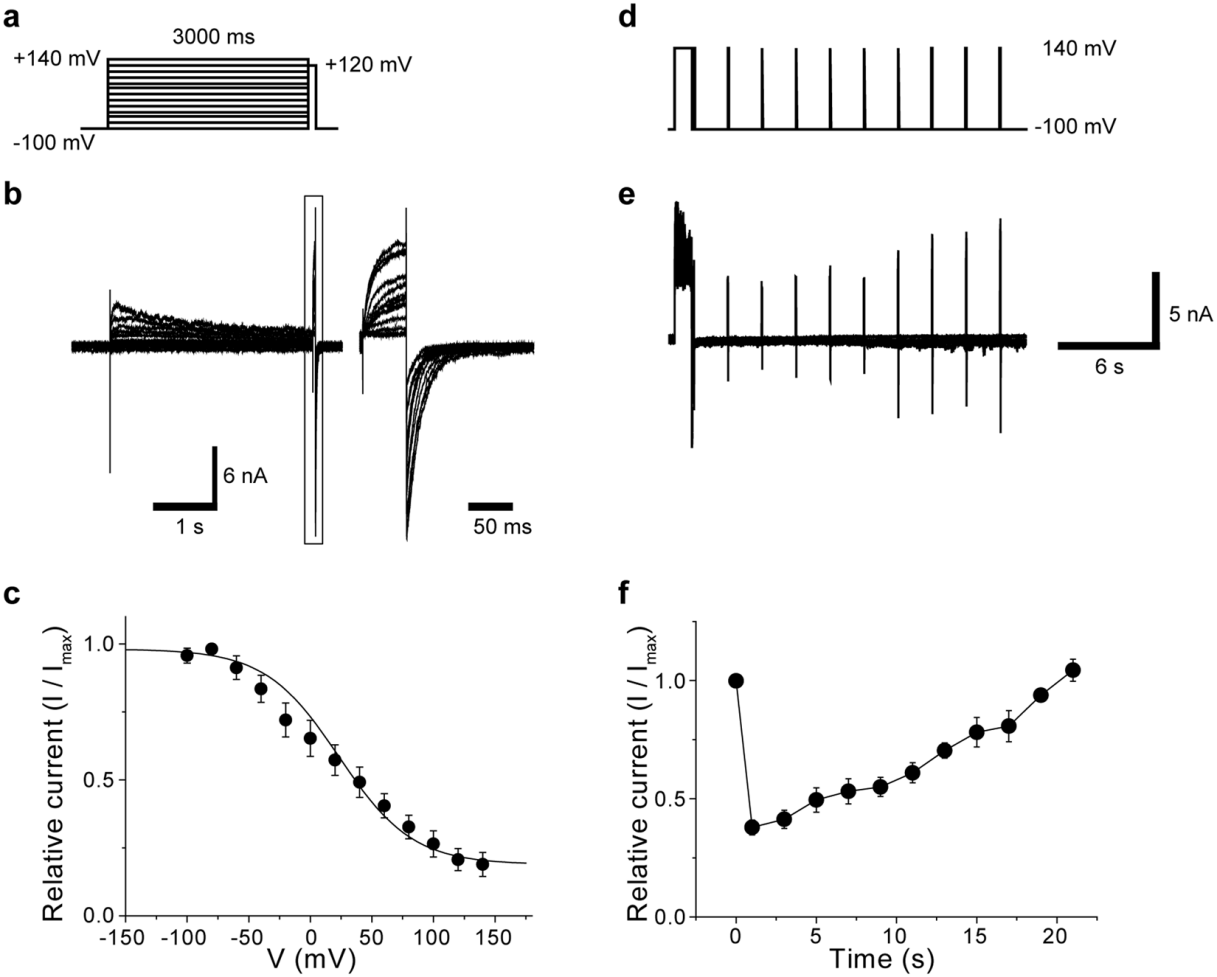
Kinetics of voltage-dependent inactivation of human PKD2L1. (**a**) Inactivation properties of PKD2L1 channels. To determine the voltage dependence of inactivation, currents were evoked by a 40 ms test pulse to +120 mV after 3 s *V*_h_ displacements (conditioning pulses) from −100 to +140 mV with 20 mV increments. (**b**) Representative superimposed current traces for steady state inactivation. (**c**) Steady state inactivation curves. Amplitudes of tail currents are normalised to the maximal peak tail amplitude and plotted against the membrane potential. Points are fitted with a Boltzmann function to yield the voltage for half-maximal inactivation (*V*_0.5_) of 14.7 mV (*n* = 6). (**d**) The time course of recovery of peak whole-cell current from inactivation. The pulse protocol was composed of two pulses, one 1 s pre-pulse from −100 to +140 mV, followed by a depolarising pulse to +140 mV with progressively prolonged durations from 0.1 to 20.1 s and then a 40 ms test pulse from −100 mV to +140 mV. The *V*_h_ was at −100 mV. (**e**) Representative superimposed current traces for recovery of whole-cell current from inactivation. (**f**) The normalised value is the result of dividing the test peak currents (*I*) by the corresponding control peak current (*I*_max_). Each symbol represents the mean value of five experiments. Data points are the means ± SEM.

Previous studies^19, 26, 27^ have found that the voltage dependence of the PKD2L1 channel unstably varies from measurement to measurement. This instability in measurement arises partly because of slow voltage-dependent inactivation at relatively depolarised holding potentials unnoticeably adopted in previous studies, as well as the difficulty of attaining the maximal channel activation, which needs extremely strong depolarisations^19^. To more precisely analyse the voltage dependency of the PKD2L1 channel under stable conditions, we designed a multi-step pulse protocol; the cell was first held at −100 mV to eliminate inactivation and subjected to different levels of depolarisation (3 s, −90 to + 140 mV) to produce varying extents of inactivation. Then, a depolarising pulse of 50 ms to + 120 mV was applied to activate the channel (Fig. 1a). The extent of inactivation was evaluated as the magnitude of inward-going tail currents at −100 mV immediately after the depolarisation to + 120 mV (Fig. 1b).

The steady-state inactivation relationship of WT PKD2L1 evaluated in this way is well described by the Boltzmann equation with a half-maximal inactivation voltage (*V*_0.5_) of + 42.2 ± 3.3 mV and slope factor *k* of + 20.3 ± 4.7 mV (Fig. 1c). The PKD2L1 channel did not appear to be fully inactivated even at very strong depolarisation levels (e.g., >  + 100 mV), whereas this channel is almost 100% available at potentials as negative as −100 mV. The relevance of this protocol was confirmed by a very slow recovery process of the channel from inactivation, which occurs in tens of seconds (Fig. 1d–f). Recovery from inactivation is > 100 times slower than the activation process ( < 100 ms; see the right inset of Fig. 1b and below). Thus, the above-used pulse protocol would not affect the extent of inactivation.

**Figure 2:**
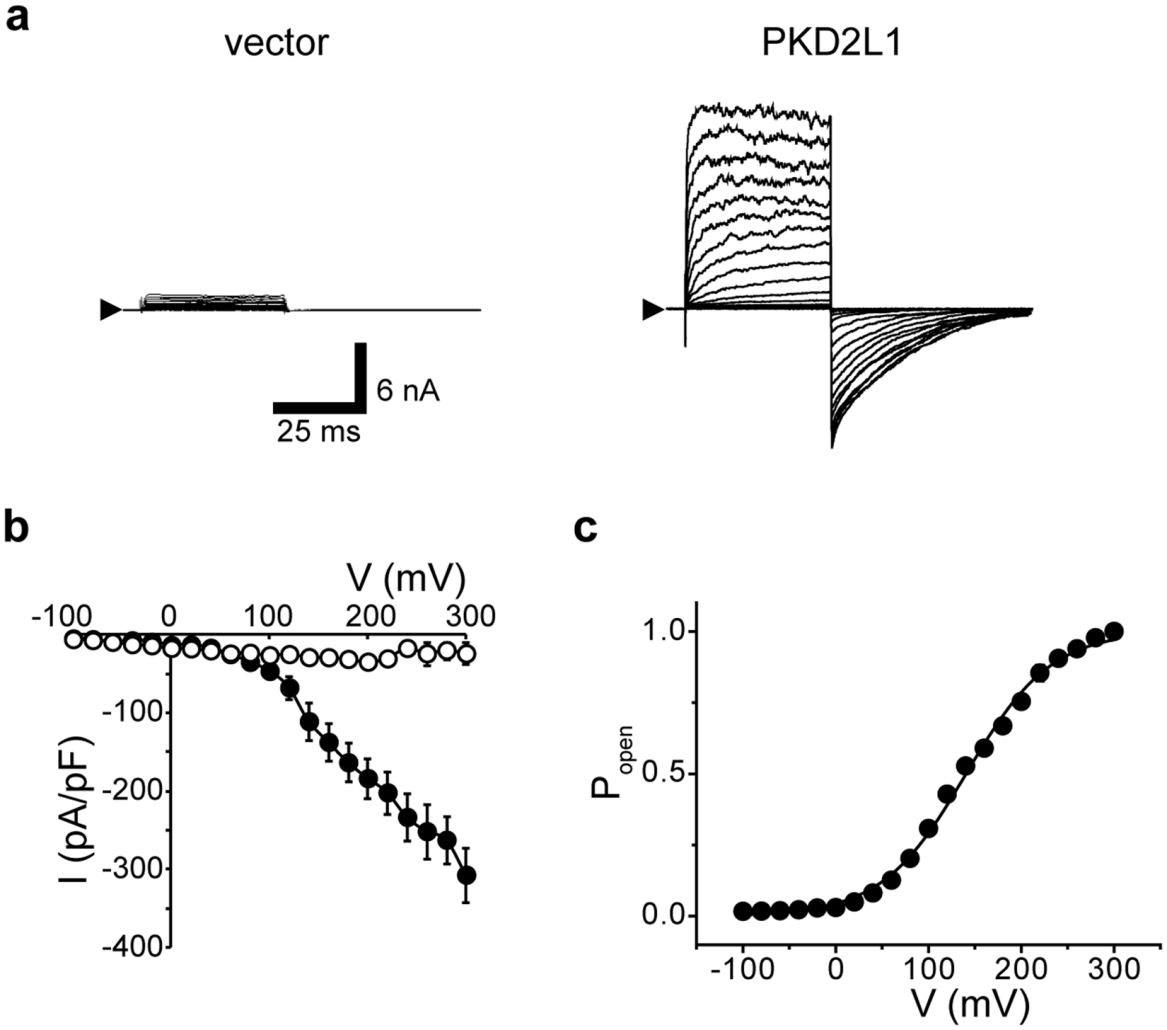
Voltage dependence of the PKD2L1 channel. (**a**) Representative whole-cell currents recorded in vector- and PKD2L1-transfected HEK293T cells. The currents elicited by application of step pulses from −100 to +300 mV in 20 mV increments at a holding potential (*V*_h_) of −100 mV. The arrowhead indicates the zero current level. (**b**) Current–voltage relationships (*I*-*V*) for instantaneous tail currents at each test pulse recorded from vector- (open circles; *n* = 7) and PKD2L1-expressing HEK293T cells (filled circles; *n* = 78). (**c**) Steady state activation curves. Amplitudes of tail currents normalised to the maximal peak tail amplitude and plotted against the membrane potential. Points are fitted with a Boltzmann function to yield the voltage for half-maximal activation (*V*_0.5_) of +132.1 mV (*n* = 71).

Based on the above results, we next investigated the voltage dependency of the activation of the PKD2L1 channel by applying a set of short (40 ms) depolarising pulses (−100 to +300 mV with a 20 mV increment, 30 s interval, holding potential of −100 mV). PKD2L1-expressing cells exhibited rapidly growing currents in response to depolarising pulses (outward-going currents in Fig. 2a). The currents were slowly deactivated upon repolarisation (inward-going tail currents in Fig. 2a). Note that the pulse duration was sufficiently long to fully activate the channel, but short enough not to cause detectable inactivation. The current–voltage (*I*-*V*) relationships constructed from the instantaneous peak and steady-state amplitudes of tail currents are shown in Fig. 2b. The steady state activation curve (*P*_open_ - *V* curve) calculated from them (for details, see the figure legend) is well fitted by the Boltzmann equation, yielding a *V*_0.5_ of + 132.1 ± 1.3 mV and a *k* of + 47.2 ± 1.1 mV (Fig. 2c).

These data demonstrate that human PKD2L1 channels have intrinsic voltage-dependency^19, 27^ that obeys classical statistical mechanics^28^ like other voltage-dependent channels.

### Mutations in S4 alter the voltage dependence

**Figure 3:**
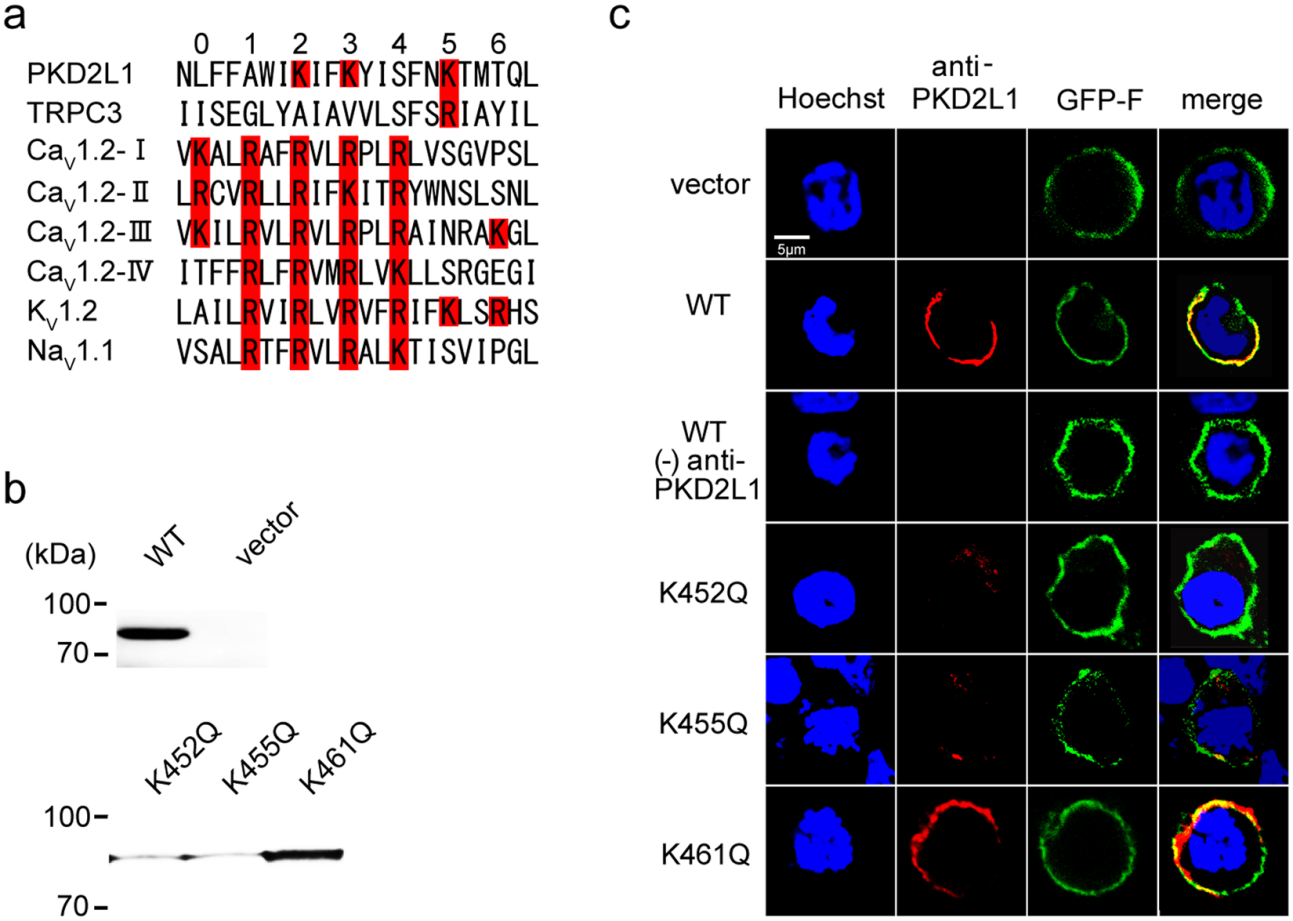
Multiple alignments of PKD2L1 and classical voltage dependent channels, and expression of S4 charge neutralised mutations of PKD2L1. (**a**) Alignment of amino acid residues of the S4 region sequences in human PKD2L1 (residues 445–466) with those in TRPC3, Ca_V_1.2, K_V_1.2 and Na_V_1.1. The residues well conserved among the aligned sequences and amino acids with positive charge are numbered and indicated in red. (**b**) Expression of PKD2L1 mutants in HEK293T cells. Western blotting analysis performed using the anti-PKD2L1 antibody. Vector transfected HEK293T cells as the negative control. (**c**) Confocal images of immunostaining with PKD2L1-specific antibody (red) reveal the localisation of PKD2L1 WT and PKD2L1 mutants (K452Q, K455Q and K461Q) at the plasma membrane. GFP-F is used as the membrane marker.

In voltage-dependent Na^+^, K^+^, or Ca^2+^ channels, the probability of channel opening (*P*_open_) is modified by the membrane potential. This is achieved through the S4 voltage sensor that detects changes in the transmembrane potential and transfers this energy to the pore domain to control channel gating^21^. Importantly, PKD2L1 has an AA sequence reminiscent of the S4 domain of a voltage-gated cation channel^23, 29^. Alignment of the S4 AA sequence of PKD2L1 with the counterparts of TRPC3, and Ca_V_1.2, K_V_1.2 and Na_V_1.1 reveals the presence of positively charged AA residues conserved in S4 (Fig. 3a). Notably, we found that the 2nd, 3rd and 5th Lys/Arg residues critical for the gating of voltage-gated channels are conserved in PKD2L1 (i.e., K452, K455 and K461; Fig. 3a). Therefore, to investigate the role(s) of these AA residues in PKD2L1 gating, we next neutralised them (Fig. 3a) by glutamine substitutions and tested their effects on the voltage sensitivity of PKD2L1. Western blot analysis confirmed robust expression of WT and the mutants K452Q, K455Q and K461Q in membrane fractions extracted from transfected HEK293T cells (Fig. 3b). The neutralised mutants K452Q and K455Q showed reduced expression levels (Fig. 3b). Results from confocal microscopy indicated that part of PKD2L1 immunoreactivity was localised in the plasma membrane of HEK293T cells transfected with either WT or the mutants (Fig. 3c).

**Figure 4:**
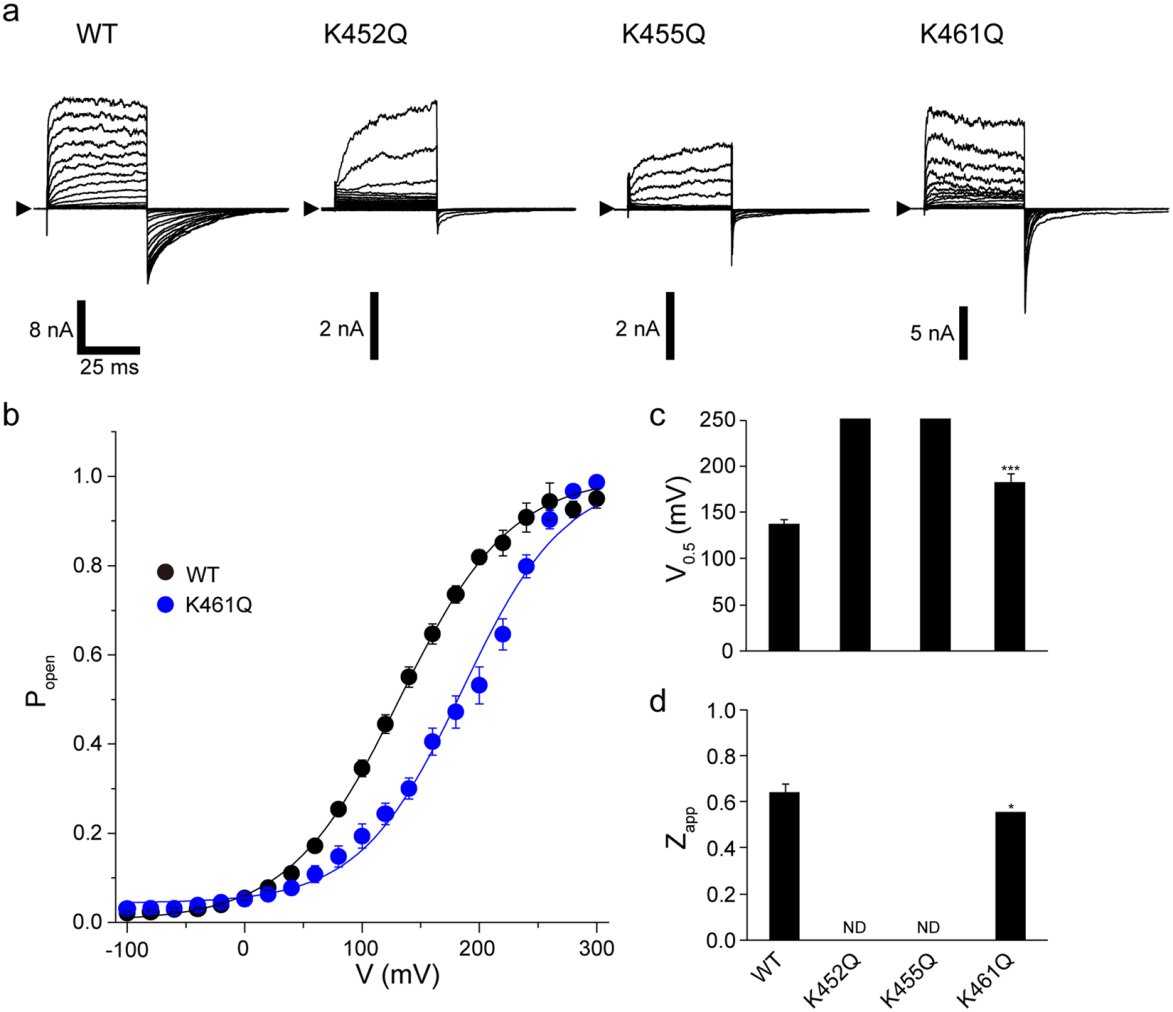
Charge neutralisation in S4 of PKD2L1. (**a**) Representative whole-cell currents in PKD2L1 WT-, K452Q-, K455Q-, or K461Q-transfected HEK293T cells. The currents elicited by application of step pulses from −100 to +300 mV in 20 mV increments at a *V*_h_ of −100 mV. (**b**) Steady state activation curves. Amplitudes of tail currents normalised to the maximal peak tail amplitude and plotted against the membrane potential. Points are fitted with a Boltzmann function to yield the voltage for half-maximal activation (*V*_0.5_) and apparent valance (*Z*_app_) values. (**c**) *V*_0.5_; WT, +138.3 ± 3.7 mV; K461Q, +182.7 ± 5.6 mV (*n* = 5–71). Data points are the means ± SEM. (**d**) *Z*_app_; WT, 0.64 ± 0.04; K461Q, 0.56 ± 0.01 (*n* = 5–71). Mutants K452Q and K455Q did not reach half-maximal activation at potentials up to +300 mV, making an estimation of *V*_0.5_ and *Z*_app_ unfeasible. Data points are the means ± SEM. ***P < 0.001. *P* values are the results of Student’s t-test.

To investigate the functional impact of charge-neutralisation, we tested the voltage dependency of mutant PKD2L1 channels using the same step-pulse protocol as above (Fig. 4a). All charge-neutralising mutants expressed in HEK293T cells formed functional channels. Amongst them, K461Q showed Boltzmann-type voltage-dependent activation similar to WT, but with a pronounced positive shift in *V*_0.5_ (Fig. 4b and c) and decreased voltage-sensitivity (i.e., reduced valence *Z*_app_; Fig. 4d). These results strongly suggest that the charged state of the S4 region is crucial for PKD2L1 gating. The K452Q and K455Q mutants did not maximally activate even upon very strong depolarisation levels (up to + 300 mV), and thus estimation of *V*_0.5_ and *Z*_app_ values was not feasible.

**Figure 5:**
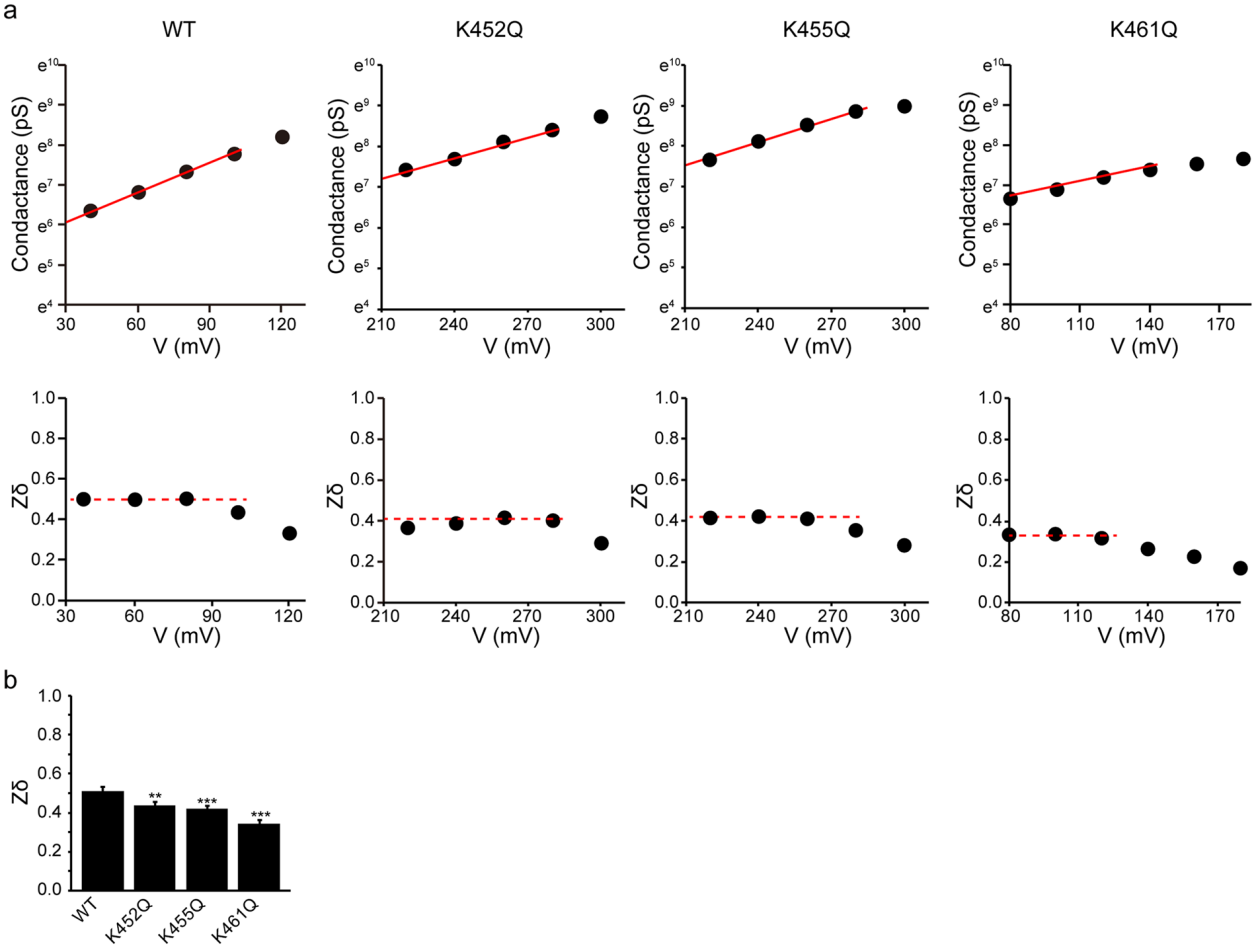
Determination of the total gating charge with the limiting slope method. (**a**) Representative chord conductance of WT and the mutants (*top*), and fittings of the slope factor *Zδ* for different voltages (*bottom*). The slope factors are fitted linearly in the marginal conductance range (*top* and *bottom*). (**b**) The effective gating charge in WT and the mutant channels. The bar depicts mean ± SEM (*n* = 10–31). **P < 0.01; ***P < 0.001. *P* values are the results of Student’s t-test.

To more explicitly estimate the number of charges involved in the activation of these channels, we next employed the ‘limiting slope analysis’^30^. Specifically, the chord conductance obtained from macroscopic currents as shown in Fig. 4 was logarithmically plotted against the membrane potential and this relationship was subjected to linear regression to assess the effective gating charge *Zδ* (Fig. 5a). The value of *Zδ*, which was 0.51 ± 0.01 for PKD2L1-WT, decreased to 0.44 ± 0.08, 0.41 ± 0.08 and 0.34 ± 0.11 for the mutants K452Q, K455Q and K461Q, respectively (Fig. 5b). Taken together, these data indicate that neutralisation of positively charged AAs at positions 452, 455 and 461 effectively reduces the number of charges apparently associated with PKD2L1 activation by membrane depolarisation. The estimates with the limiting slope analysis do not differ much from those obtained from the Boltzmann curves (*Z*_app_; Fig. 4b).

### Mathematical reconstruction of macroscopic PKD2L1 currents

Simulation with mathematical models is often instrumental in defining the gating kinetics of ion channels over extreme voltage ranges where electrophysiological measurements are practically unfeasible^31^. Taking advantage of such an approach, we next constructed a mathematical model appropriately describing the voltage-dependent gating kinetics of the WT-PKD2L1 channel. The model was then modified to fit the voltage-dependence of mutant PKD2L1 channels that could not be fully activated even by extreme depolarisation (Fig. 5).

**Figure 6:**
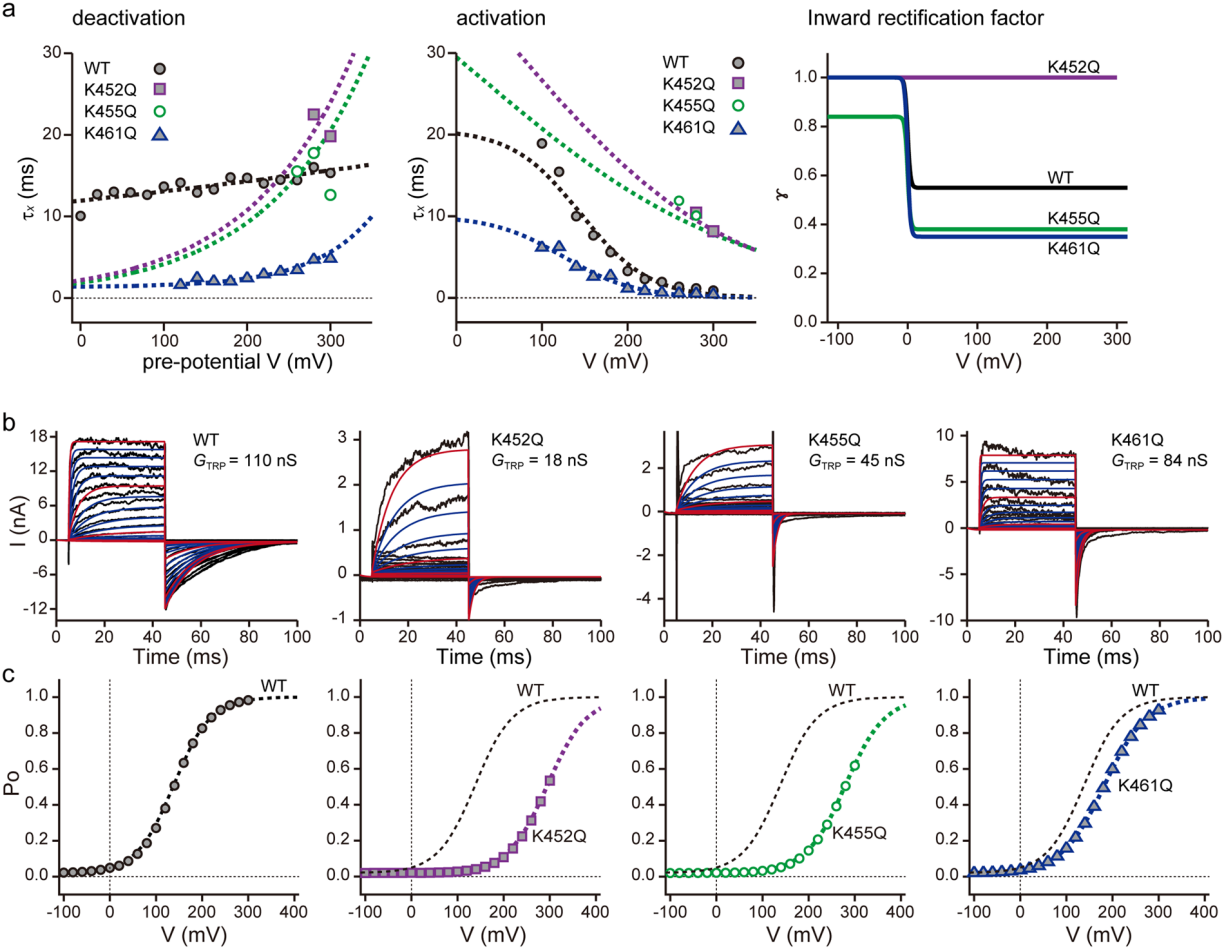
Parameters of activation and deactivation kinetics from experimental data. (**a**) (*left panel*) The deactivation kinetics of the PKD2L1 channels and mutants are obtained by deactivation kinetics of tail currents. The fitting for deactivation data is determined by nonlinear least-squares fitting. (*centre panel*) The activation kinetics of PKD2L1 channels and mutants are computed by activation kinetics of outwardly whole cell currents. The fitting parameter for activation kinetics is determined by nonlinear least-squares fitting. (*right panel*) The predicted values of inward rectification factors (*γ*). (**b**) Simulation of whole-cell currents in WT and mutants in voltage clamp measurements. A voltage clamp step from −100 to +300 or +400 mV in 20 mV increments at a *V*_*h*_ of −100 mV was applied to the macroscopic PKD2L1 current (*I*_PKD2L1_) model. (**c**) The relationship between total conductance (*P*_O_) and membrane voltage.

The time constants of activation and deactivation were obtained by voltage-jump experiments by fitting the time courses of current growth or decline with appropriate mathematical functions (Fig. 6a). Then, rate constants (Fig. 4) calculated from the time constants and *P*_open_ values were used as the initial estimates for numerical optimisation, constrained by microscopic reversibility and subjected to sensitivity analysis. After optimising the mathematical formulation of the rate constants for open and close transitions, macroscopic PKD2L1 currents activated by respective depolarising pulses were numerically calculated, according to eqs 1–5.

As shown in Fig. 6b and c, simulated currents reproduced reasonably the observed voltage-dependence of PKD2L1 currents in WT, as well as in the K452Q, K455Q and K461Q mutants. However, it was necessary to introduce an additional scaling factor (*SF*) to adjust the chord conductance for respective mutant channels, depending on the direction of ionic flow (Fig. 6a, *right panel*). As a result, *G*_TRP_ in eq. 1 was re-written as *G*_TRP_ = *G*_TRP,max_ *·SF* (*V*_m_), where *G*_TRP,max_ is the maximal conductance for each channel and *SF*(*V*_m_) is a function of the membrane potential.

**Figure 7:**
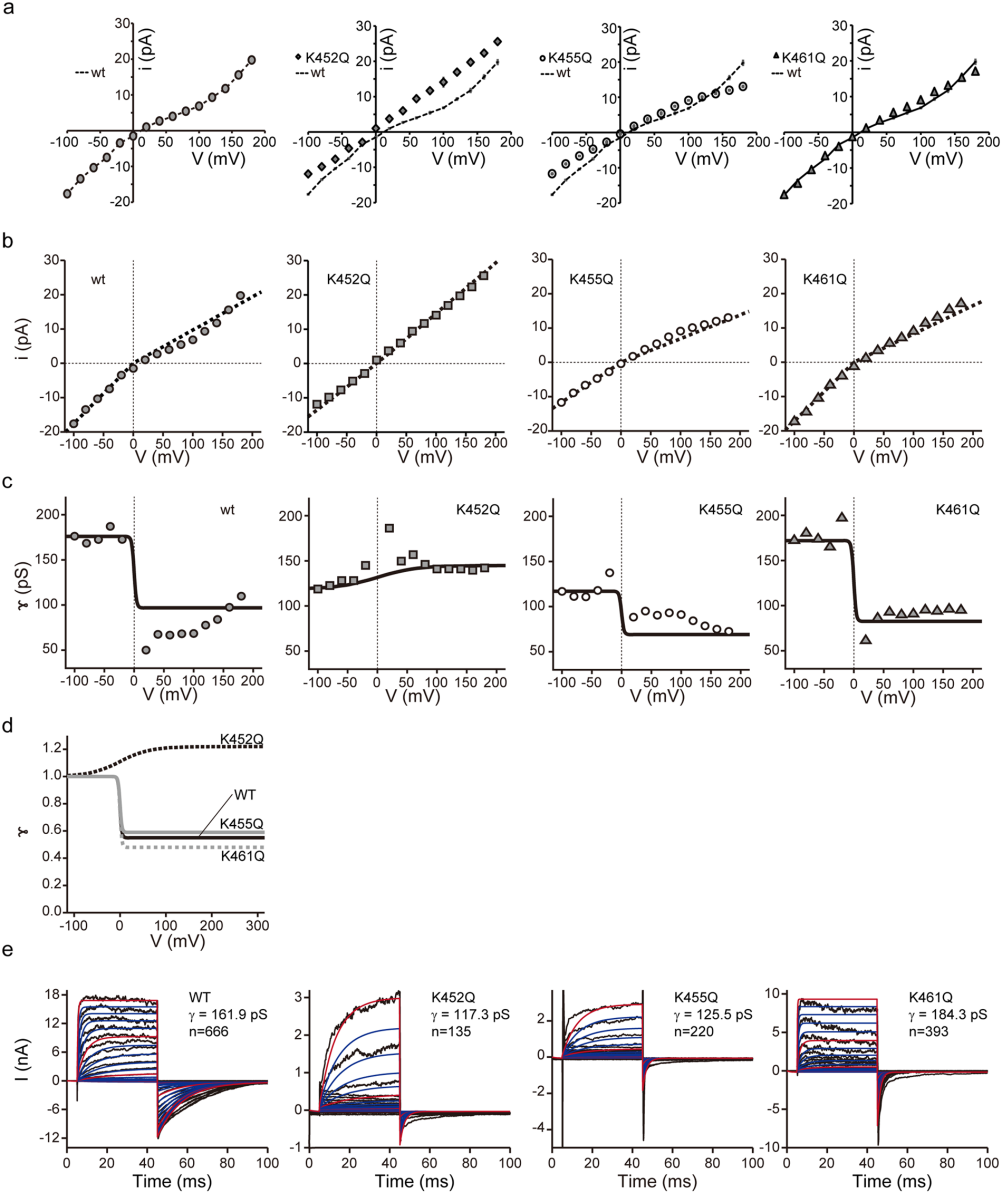
Single channel recordings of the PKD2L1 channels. (**a**) Current–voltage relationships (*i*-*V*) for constitutively active currents elicited by a ramp pulse from −100 mV to +180 mV after reducing the number of available channels by inactivation (holding potential; 0 mV) recorded for PKD2L1-WT (filled circles; *n* = 15), -K452Q (circles; *n* = 7), -K455Q (triangles; *n* = 7) and -K461Q (squares; *n* = 10) expressing HEK293T cells. (**b**) Calculated *i*-*V* relationships were well fitted to the experimental value. Each type of symbol represent experimental values from (**a**). The dotted line shows the predicted data from Fig. 6a (*right panel*). (**c**) Chord conductance calculated from the predicted inward rectification factor are well fitted to the experimental values. Each type of symbol represents experimental values from (**a**). The dotted line shows the calculated values from the predicted inward rectification factors. (**d**) Inward rectification factor (*γ*) obtained from (**c** and **e**). Macroscopic current simulations are modified by experimental values from (**d**).

One plausible mechanism for this apparent voltage-dependence of maximal conductance is the intrinsic rectifying property or partial opening (or dilation) of an ion conductive pore^32–34^. To examine this possibility more directly, we next measured the unitary conductance (*γ*) of constitutively active WT and mutant PKD2L1 channels by means of single channel recording. As summarised in Fig. 7a and Supplementary Fig. S7, the reversal potential of the unitary current does not change for the mutants. However, the value of *γ* is larger for inward than outward directions in WT and the K461Q mutant (Fig. 7c and Table 1). This disparity of *γ*, i.e., inward-rectifying property, is markedly attenuated in K452Q and K455Q mutants. In comparison with WT, the *γ* value is larger for the outward direction in K452Q but decreased for both directions in K455Q (Fig. 7b and d). By incorporating these results in the mathematical model, we could precisely reproduce the time-dependent changes of WT and mutant PKD2L1 currents observed in patch clamp experiments (Fig. 7e).

**Table 1:** Single channel conductance of PKD2L1 and mutants.

Slope conductance	Inward (-100~40 mV)	n	Outward (40~140 mV)	n
WT	161.9 ± 4.7 pS	15	102.4 ± 5.0 pS	16
K452Q	117.3 ± 5.5 pS	9	137.8 ± 9.6	7
K455Q	125.5 ± 6.2 pS	7	77.0 ± 3.0 pS	9
K461Q	184.3 ± 9.7 pS	10	85.9 ± 5.7 pS	15

The above results imply that the three positively charged K452, K455 and K461 residues in the putative S4 region of the PKD2L1 channel may differentially contribute to its voltage-dependent activation through at least two distinct mechanisms, i.e., alteration of *P*_open_ and rectification of unitary ionic flow or the voltage-dependence of *γ*.

## Discussion

PKD2L1 has been reported to form a nonselective cation channel when it is expressed in *Xenopus* oocytes^35–39^, planar lipid bilayers^40^ or mammalian cells^19, 26, 27, 41^. Since its maximum activation requires extremely strong depolarisation and inactivation proceeds time-dependently, it was technically difficult to accurately evaluate the voltage-dependent kinetics of PKD2L1^19^. However, by applying the optimised voltage-pulse protocol, we could clearly demonstrate three important features of this channel. First, the expressed PKD2L1 channel has constitutive activity and the membrane potential (i.e., holding potential) only modulates the channel by changing the extent of slow inactivation. Second, the voltage-dependent development/decline (or activation/deactivation) of the PKD2L1 channel by depolarisation/repolarisation follows fast kinetics of tens of milliseconds, while its inactivation/recovery requires more than 100-fold longer time courses (Figs 1 and 2). Third, the large unitary conductance of the PKD2L1 channel (162 pS) is inward rectifying, but this property is independent of divalent cation blockade or other conduction block mechanisms (Table 1). This result is consistent with those of previous studies^19, 27^. These findings together suggest that: (1) membrane depolarisation *per se* is not mandatory to activate the PKD2L1 channel but strongly modulatory, and, when it prolongs, induces the slow inactivation of the channel; and (2) ion conduction through the pore of PKD2L1 may also be intrinsically voltage-dependent.

### Comparison of PKD2L1 with other voltage-dependent channels

The PKD2L1 channel has constitutive activity around the resting membrane potential, but it is voltage-dependently enhanced, which follows a Boltzmann-type relationship with an apparent *V*_0.5_ of + 132.1 ± 1.3 mV and a *k* of + 47.2 ± 1.1 mV (Fig. 2c). However, these values are largely variable from study to study. It seems likely that this discrepancy arises from variable extents of voltage-dependent inactivation involved therein. Indeed, by applying a sufficiently long and negative holding potential (−100 mV), we could successfully remove inactivation and reproducibly obtain the voltage-dependent gating parameters (Fig. 1).

In most isoforms of TRP channels, the midpoint of the voltage-dependent activation curve is very positive under normal conditions. This makes the channel activity very small at physiological membrane potentials, as observed in TRPV1^17, 42^, TRPV3^43^, TRPM3^44^, TRPM4^7^, TRPM5^45^ and TRPM8^17, 42^ channels. However, application of specific ligands or modulators often causes a dramatic negative shift of the activation curve, inducing substantial channel activities at physiological membrane potentials. This holds true for the PKD2L1 channel, in which the activation curve undergoes a significant negative shift by acidic extracellular pH, hypotonicity-induced cell swelling, or elevated extracellular Ca^2+^ concentration^19, 27^.

The voltage sensor domain of the voltage-gated K channel (K_V_) is localised to the fourth transmembrane S4 bearing an array of positive charges, and conformational change is transmitted via the S4–S5 linker to gate the pore domain^21^. A homologous structure exists in weakly voltage-dependent (or modulated) channels such as TRP channels and some of them (e.g., TRPM8) appear to gate through a similar voltage-dependent mechanism^46^. In our present study, the neutralising mutations of positively charged AA residues K452Q, K455Q and K461Q in S4 weakened the voltage dependence of PKD2L1, accompanying a positive shift of the *G*-*V* curve (Fig. 4) with reduced gating charge (Figs 4d and 5). The total gating charge was previously assessed for several voltage-sensitive TRP channels by the limiting slope method. However, the obtained values were much smaller than those of voltage-gated channels (e.g., ~7 for Kv) and well below 1.0, e.g., 0.89 (TRPM8)^46^, 0.7 (TRPM4)^7^ and 0.375 (TRPA1)^20^. We also obtained a similar value for the total gating charge of PKD2L1 (0.51; Fig. 5). Importantly, however, the gating charge was reduced efficiently by single mutations in the putative S4 domain of the PKD2L1 channel, i.e., K452Q, K455Q or K461Q (reduced by 0.07–0.17), which resulted in significantly reduced voltage sensitivity. This strongly indicates that these lysine residues may somehow contribute to the voltage sensing/gating of the PKD2L1 channel.

The positive charges in the S4 region are usually formed as the cluster of arginine residues. The rationale behind it may be the energetically lower cost for arginines (by about 1.0 kcal mol^−1^) than lysines to transfer their diffuse positive charges from water to the lipid phase^47^. Because PKD2L1 has three uniquely lined lysines in S4, the activation of its voltage sensor may require an additional 3 kcal per one subunit. This could explain in part why the *V*_0.5_ value of the PKD2L1 channel is more positive (by ~100 mV) than those of typical voltage-dependent channels.

### Possible mechanisms involved in the voltage dependence of PKD2L1

To gain more insights about the role of the predicted voltage sensor of PKD2L1, we performed homology modelling based on two atomic structures recently resolved by CryoEM: PKD2 (PDBID: 5T4D)^25^ and Na_V_Ab (PDBID: 3RVY)^48^. The former channel has the highest degree of overall AA sequence identity (61.63%) to PKD2L1 among the polycystin subfamily. The structure of PKD2 in a lipid bilayer resolved at 3.0 Å is presumed to represent a closed or resting state characterised by two narrowed parts in the selectivity filter region (due to L641, G642, D643) and lower gate (due to L677 and N681) that restrict ionic flow. Na_V_Ab, a bacterial tetrameric voltage-gated Na channel, appears to have similarities to PKD2L1 in the arrangement of critical AA pairs involved in electrostatic and hydrogen-bonding interactions during voltage sensor movement^21, 22^, as shown by AA alignment (Supplementary Fig. S1). The resolved structure of Na_V_Ab provides a pre-open state that is undergoing partial transition from resting to fully-open configurations.

In the modelled structure of PKD2L1 based on PKD2, which is presumed to show the resting state, the side chains of K452 in S4 (K405 in PKD2) and D390 in S3 (D343 in PKD2) are located sufficiently close (4.3 Å) to interact with each other, and those of K455 in S4 (K408 in PKD2) and D390 in S3 (D343 in PKD2) are even in closer apposition (3.6 Å). The locations of these two AA pairs also appear to exist in Na_V_Ab (R2 and D80, R3 and D80). At the position of D390 in PKD2L1, two other adjacent negative AA residues E369 (6.4 Å) and E373 (5.1 Å) of S2 may also contribute to counter act K455 (K408 in PKD2). (Supplementary Fig. S2)

Homology modelling by substituting the S2–S4 domains of Na_V_Ab with those of PKD2L1 or its mutant K452Q, which is presumed to represent a pre-activated state (Supplementary Figs S3 and S4), gives a different picture. In the WT-PKD2L1 model, the upward-translocated and clockwise-rotated voltage sensor is stabilised via both electrostatic and hydrogen-bonded interactions of K452 (with V439: 3.44 Å), K455 (with S458: 2.81 Å) and K461 (with E413: 2.98 Å, D430: 2.97 Å, and N427: 2.90 Å). In the modelled structure of the K452Q-mutant (Supplementary Fig. S4), however, these three lysine residues interact differently forming new pairs: K455 and S458 (3.04 Å), K461 and E413 (2.96 Å), and K461 and N427 (3.40 Å). The neutralised K452 by glutamine substitution no longer contributes to voltage sensor stabilisation. Probably because of these changes voltage-dependent translocation/rotation of the voltage sensor of the K452Q mutant would be greatly suppressed. In the absence of K452-mediated hydrogen bond formation, the rotation of the voltage sensor is restricted by ~12° compared with that of the WT. This suppression may cause a more positive shift of the *G*-*V* curve in the K452Q mutant than in WT.

Markedly attenuated voltage sensor movement in the K452Q mutant may also affect the inward rectifying property of unitary conductance (Fig. 7a). By analysing and comparing the pore structures of PKD2-based PKD2L1 and PKD2L1-Na_V_Ab hybrid models with the HOLE software, it is suggested that, compared with the resting state, the pore diameter of the pre-activated PKD2L1 channel may be greatly distended, in particular at the position of F502 (which corresponds to T224 in Na_V_Ab). This AA is located at the central cavity near the entrance to function as a selectivity filter and thus might influence the ionic flow. (Supplementary Fig. S5) Consistent with this concept, it has been suggested that a wide inner cavity of BK channels could provide a low access-resistance pathway allowing its high single channel conductance, and that F380 located at the inner cavity is a critical determinant of potassium permeation, mutation to Ile or Trp has been shown to reduce greatly the conductance^49^. Therefore, it is conceivable that voltage-dependent dilation of the central cavity (and translocation of F502) affects the inward rectifying property of the PKD2L1 channel. This structural change might be more prominent in the K452Q mutant, which shows an almost doubled unitary conductance for the outward direction. Obviously, further systematic mutational and structural studies are required to corroborate these possibilities.

In conclusion, we have found that positive charges in S4 critically affect the voltage-dependence of not only the open probability (*P*_open_) but also the unitary conductance (*γ*) of the PKD2L1 channel by combined applications of patch clamp experiments and numerical model simulations. The present theoretical approach can be generalised to channels that possess non-classical voltage-dependent properties.

## Methods

### Molecular cloning and plasmid construction

Human PKD2L1 (GenBank accession No. NM_016112.2) was cloned from human brain, whole marathon-ready cDNA (BD Biosciences, San Jose, CA, USA) by applying a PCR-based approach designed to contain the untranslated leader sequence from the alfalfa mosaic virus^50^ and consensus sequence from the translation initiation^51^, and was subcloned into the expression vector pCI-neo (Promega, Madison, WI, USA). PKD2L1 glutamine mutants were constructed from PKD2L1-pCI-neo using overlap extension PCR^52^. The primer pairs used for K452Q, K455Q, and K461Q are summarised in Table 2.

**Table 2:** Primer sequences used for overlap extension PCR in producing mutants.

Genes	Mutants	Mutation primer sequences (5′ → 3′)	Restriction sites used for cloning into PKD2L1-pCIneo
PKD2L1	K452Q	for: CTCTTCTTCGCCTGGATCGAGATATTCAAGTACATCAGCTTCAAC	BamHI/NotI
		rev: GTTGAAGCTGATGTACTTGAATATCTCGATCCAGGCGAAGAAGAG	
	K455Q	for: CTGGATCAAGATATTCGAGTACATCAGCTTCAACAAAACCATGAC	
		rev: GTCATGGTTTTGTTGAAGCTGATGTACTCGAATATCTTGATCCAG	
	K461Q	for: CAAGTACATCAGCTTCAACGAAACCATGACCCAGCTCTCCTCCAC	
		rev: GTGGAGGAGAGCTGGGTCATGGTTTCGTTGAAGCTGATGTACTTG	

### Cell cultures and cDNA expression

HEK293T cells were grown in Dulbecco’s modified Eagle’s medium (DMEM) supplemented with 10% fetal bovine serum, 30 units/ml penicillin G and 30 μg/ml streptomycin under a 95% air −5% CO_2_ atmosphere at 37 °C. HEK293T cells in 3.5 cm dishes were transfected 24 h after plating with the plasmids pEGFP-N1 (Clontech Laboratories, Palo Alto, CA, USA) and pCI-neo (Promega) for vector-transfected (vector) cells, or pEGFP-N1 and PKD2L1-pCI-neo for PKD2L1-transfected (PKD2L1, WT) cells. Lipofectamine 2000 (Invitrogen, Carlsbad, CA, USA) was used for transfections according to the manufacturer’s instructions. Electrophysiological measurements, western blot analysis and immunofluorescence staining were performed 24–48 h after transfection. For immunofluorescence staining, HEK293T cells were co-transfected with recombinant plasmids and pEGFP-F (Clontech Laboratories) as the marker for plasma membrane localisation.

### Immunofluorescence staining

Rabbit antiserum to human PKD2L1 was raised against the residues 791–805 (ERRLSRGEIPTLQRS). Cells on coverslips were fixed with 4% (v/v) formaldehyde in phosphate-buffered saline (PBS) for 30 min at 4 °C, washed with PBS three times and then permeabilised with 0.1% (v/v) Triton X-100 in PBS for 10 min at room temperature. After washing in PBS three times, the coverslips were incubated with 10% normal goat serum (NGS) for 1 h at room temperature. The cells were then incubated overnight at 4 °C with the polyclonal antibody (1/1000), diluted in 1% NGS in PBS, and incubated for 1 h with the Cy3-conjugated anti-rabbit IgG (1/4000). The coverslips were sealed with Perma Fluor Aqueous Mounting Medium (Thermo Shandon, Pittsburgh, PA, USA) to prevent evaporation and stored at 4 °C before imaging. The fluorescence images were acquired with a FV500 confocal laser scanning microscope (Olympus, Tokyo, Japan) equipped with a krypton/argon ion laser. The fluorescence images were acquired with a confocal laser-scanning microscope using the 488-nm line of an argon laser for excitation and a 505-nm to 525-nm band-pass filter for emission, or the 543-nm line of a HeNe laser for excitation and a 560-nm long-pass filter for emission.

### Electrophysiology

After co-transfection with pEGFP-N1 and the plasmids containing the cDNA for WT, vector, K452Q, K455Q, or K461Q, coverslips with cells were placed in dishes containing the solutions. Currents from cells were recorded at room temperature (22–27 °C) using patch-clamp techniques of the whole-cell mode, with an EPC-9 (HEKA Electronics, Lambrecht, Germany) or an Axopatch 200B (Axon Instruments/Molecular Devices, Union. City, CA, USA) patch-clamp amplifier. The patch electrodes prepared from borosilicate glass capillaries had a resistance of 3–5 MΩ for whole cell recordings. Current signals were filtered at 5 kHz with a four-pole Bessel filter and digitised at 10 or 20 kHz. PULSE (version 8.8; HEKA Electronics) or pCLAMP (version 10.0.2; Axon Instruments/Molecular Devices) software was used for command pulse control, data acquisition and analysis. For whole cell recordings, series resistance was compensated (to 70–80%) to minimise voltage errors. The external solution contained 100 mM NaCl, 2 mM Ca-gluconate and 10 mM 4-(2-hydroxyethyl)-1-piperazineethanesulfonic acid (HEPES) (pH 7.4 adjusted with NaOH, and osmolality adjusted to 320 mmol/kg with D-mannitol). The pipette solution contained 100 mM Cs-aspartate, 5 mM 1,2-bis(o-aminophenoxy)ethane-N,N,N′,N′-tetraacetic acid (BAPTA), 2.3 mM Ca-gluconate, 2 mM Mg_2_ATP, 2 mM Mg_2_SO_4_, 1 mM MgCl_2_ and 10 mM HEPES (pH 7.4 adjusted with CsOH, and osmolality adjusted to 300 mmol/kg with D-mannitol). The free Ca^2+^ concentration was 100 nM calculated with the CaBuf software^53^. Data was analysed using IGOR (Wavemetrics, Lake Oswego, OR, USA) and Origin (OriginLab Corp., Northampton, MA, USA) software. The tail currents measured during the current–voltage relationship were fitted using the Boltzmann equation: *I/I*_max_ = 1/(1 + exp((*V*_0.5_ − *V*_m_)/*k*)), where *V*_m_ is the membrane potential, *V*_0.5_ is the potential to give a half-value of conductance, *k* is the slope factor and *I*_max_ is the saturating current. The apparent channel open probability (*P*_open_) was determined as *G/G*_max_, where *G* represents the steady state conductance and *G*_max_ represents the saturating conductance measured at strongly depolarised potentials. Accordingly, we fitted activation curves using the Boltzmann function: *P*_open_ = 1/(1 + exp(−*z*(*V* − *V*_0.5_)/*k*_B_ *T*)) where *z* is the gating charge (in elementary charge units: e_o_ = 1.6 × 10^−19^ C), *V* is voltage, *V*_0.5_ is the voltage for half-maximal activation, *k*_B_ is the Boltzmann constant (1.38 × 10^−23^ J/K) and *T* is the absolute temperature. To obtain a gating charge, we used the limiting slope conductance method^28^. Briefly, the valence of the apparent single-gate charge for activation (Zδ) was calculated from the *k*_a_ value, using the equation Zδ = *k*_*a*_ *k*_*B*_ *T*/*e*. The *k*_a_ value was obtained from conductance versus voltage relationships.

### Western blotting analysis

After 36 h of transfection, HEK293T cells were solubilised in radioimmunoprecipitation assay (RIPA) buffer (pH 8.0) containing 0.1% sodium dodecyl sulfate (SDS), 0.5% sodium deoxycholate, 1% Nonidet P40, 150 mM NaCl, 50 mM Tris-HCl, 1 mM phenylmethylsulfonyl fluoride (PMSF) and 10 μg/μl leupeptin, and then centrifuged at 17,400 × *g* for 20 min. Immunoprecipitated proteins were fractionated by 7.5% SDS polyacrylamide gel electrophoresis (PAGE) and electrotransferred onto a polyvinylidene fluoride (PVDF) membrane. The blots were incubated with an anti-PKD2L1 antibody and stained using the Enhanced Chemiluminescence system (Thermo Scientific, Rockford, IL, USA).

### Formulations of kinetic properties for PKD2L1 currents and simulation protocol

To simulate the voltage dependence in PKD2L1, we first constructed the macroscopic PKD2L1 current (*I*_PKD2L1_) model, which is restricted to the activation-deactivation kinetics, because in a short period of time (<100 ms) slow transition to inactivation is almost negligible (Fig. 1). The *I*_PKD2L1_ was defined as

1 $${I}_{{\rm{PKD}}2{\rm{L}}1}={G}_{{\rm{TRP}}}\cdot {x}_{i}\cdot ({V}_{m}-11.64)$$

where *G*_TRP_ is the maximal conductance (nS/pF), and *x*_*i*_ is the activation variable in *I*_PKD2L1_. The reversal potential of 11.64 mV is obtained from macroscopic PKD2L1 current-voltage relationships (Supplementary Fig. S6). The voltage-dependence of the activation variable in the *I*_PKD2K1_ was calculated with the following first-order differential equation

2 $${{\rm{dx}}}_{{\rm{i}}}/{\rm{d}}t=({x}_{\infty ,i}-{x}_{i})/{\tau }_{\iota }$$

where *i* represents the channel type, where the channel type can be PKD2L1WT, K452Q, K455Q, or K461Q, *x*_*i*_ is the state variable of *I*_PKD2L1_ for the channel types of *i*, *x*_∞,*i*_ is the steady-state value of *x*_*i*_, and *τ*_ι_ is the time constant for *x*_*i*_. From our experimental voltage-clamp measurements, *x*_∞,*i*_ was fitted as a single Boltzmann function:

3 $${x}_{\infty ,{\rm{WT}}}=0.02+0.98/(1+\exp (-z({V}_{{\rm{m}}}\,\mbox{--}\,{V}_{0.5})/{k}_{{\rm{B}}}T)).$$

For *x*_∞,*i*_ in each type of channel, the half-maximal activation voltages and slope factors are listed in Table 3. Each time constant *τ*_*ι*_, for *i* = WT, K452Q, K455Q and K461Q, was derived from current activation (typically *V*_m_ greater than 100 mV) and current deactivation experiments (typically *V*_m_ less than 0 mV), and was expressed as functions of *V*_m_ as follows:

4 $$\begin{array}{c}{\rm{For}}\,{V}_{{\rm{m}}}\ge 0,{\tau }_{{\rm{WT}}}=20.7/(1+\exp (0.025({V}_{{\rm{m}}}-145)));\\ {\tau }_{{\rm{K}}452{\rm{Q}}}=60.6/(1+\exp (0.008{V}_{{\rm{m}}}-70));\\ {\tau }_{{\rm{K}}455{\rm{Q}}}=54.6/(1+\exp (0.0065({V}_{m}-25)));\\ {\rm{and}}\\ {\tau }_{{\rm{K}}461{\rm{Q}}}=10.12/(1+\exp (0.0237({V}_{{\rm{m}}}-125.2))).\end{array}$$

5 $$\begin{array}{c}{\rm{For}}\,{V}_{{\rm{m}}} < 0,{\tau }_{{\rm{WT}}}=10.9\exp (\mbox{--}\,0.001{V}_{{\rm{m}}});\\ {\tau }_{{\rm{K}}452{\rm{Q}}}=1.7\exp (-0.008({V}_{{\rm{m}}}+69.1));\\ {\tau }_{{\rm{K}}455{\rm{Q}}}=1.6\exp (-0.008({V}_{{\rm{m}}}+80.75));\\ {\rm{and}}\\ {\tau }_{{\rm{K}}461{\rm{Q}}}=1.33+0.16\exp (-0.014{V}_{{\rm{m}}}).\end{array}$$

**Table 3:** Half-maximal activated voltage and slope factor estimated from the voltage-clamp experiments in PKD2L1 for each channel type.

Type	*V*_0.5_ (mV)	z/k_B_T
WT	138.3	0.0249
K452Q	295.0	0.0234
K455Q	280.0	0.0233
K461Q	182.7	0.0217

The values in eqs 4 and 5 were determined from our experimental data by nonlinear least-squares fitting.

All simulations for calculating *I*_PKD2L1_ for each clamped voltage pulse with 40 ms duration were performed using the forward Euler method with a 0.01 ms time step. The activation variable (*x*_*i*_) was set equal to zero as an initial condition. Simulations were encoded in C/C +  + , and run on an IBM-compatible computer with the Intel ICC compiler version 15.0.1.

### Bioinformatics

Sequence alignments were done using the Clustal Omega multiple sequence alignment tool^54^ with the following Uniprot codes: PKD2L1 (Q9P0L9), PKD2 (Q13563), TRPC3 (Q13507), Ca_V_1.2 (Q13936), K_V_1.2 (P16389), Na_V_1.1 (P35498) and Na_V_Ab (A8EVM5). Structural alignments and figures were generated using SWISS-MODEL^55^. The following PDB entries were used for structural comparisons: Na_V_Ab (3RVY) and PKD2 (5T4D). Inner pore measurements were calculated using the HOLE suite of programmes^56^ and visualised with the Visual Molecular Dynamics (VMD)^57^ or PyMOL^58^ programs. Hydrogen bonding analysis was used using the Hydrogen Bond Calculation program^59^.

### Statistical analyses

All data are expressed as means ± SEM. We accumulated the data for each condition from at least three independent experiments. The statistical analyses were performed using Student’s *t*-test. A value of *P* < 0.05 was considered significant.

### Data availability

All relevant data are included within the paper.

## Electronic supplementary material

Supplementary Information
