# Supplementary material for: Integrative Approach with Electrophysiological and Theoretical Methods Reveals a New Role of S4 Positively Charged Residues in PKD2L1 Channel Voltage-Sensing
Source: Sci Rep. 2017 Aug 29;7:9760. doi: 10.1038/s41598-017-10357-3 (PMC5575081; doi:10.1038/s41598-017-10357-3)
Supplement: Supplementary file 1 — Supplementary Information [file 41598_2017_10357_MOESM1_ESM.pdf]

# Supplementary information

## Integrative approach with electrophysiological and theoretical methods reveals a new role of S4 positively charged residues in PKD2L1 channel voltage-sensing

Tomohiro Numata[1,2*], Kunichika Tsumoto[3], Kazunori Yamada[4,5], Tatsuki Kurokawa[2], Shinichi Hirose[6], Hideki Nomura[7], Mitsuhiro Kawano[4], Yoshihisa Kurachi[3], Ryuji Inoue[1*] & Yasuo Mori[2*]

[1]Department of Physiology, Graduate School of Medical Sciences, Fukuoka University, 7-45-1 Nanakuma, Johnan-ku, Fukuoka, Fukuoka 814-0180, Japan. [2]Laboratory of Molecular Biology, Department of Synthetic Chemistry and Biological Chemistry, Graduate School of Engineering, Kyoto University, Katsura, Nishikyo-ku, Kyoto, Kyoto 615-8510, Japan. [3]Department of Pharmacology, Graduate School of Medicine, Osaka University, 2-2 Yamada-oka, Suita, Osaka 565-0871, Japan. [4]Division of Rheumatology, Department of Internal Medicine, Kanazawa University Graduate School of Medicine, 13-1 Takara-machi, Kanazawa, Ishikawa 920-8641, Japan. [5]Department of Advanced Research in Community Medicine, Kanazawa University Graduate School of Medical Sciences, 13-1 Takara-machi, Kanazawa, Ishikawa 920-8641, Japan. [6]Department of Pediatrics School of Medicine, Fukuoka University, 7-45-1 Nanakuma, Johnan-ku, Fukuoka, Fukuoka 814-0180, Japan. [7]Department of General Medicine, Kanazawa University Hospital, 13-1 Takara-machi, Kanazawa, Ishikawa 920-8641, Japan. Correspondence and requests for materials should be addressed to T.N (email: numata@ fukuoka-u.ac.jp), R.I (email: inouery@ fukuoka-u.ac.jp) and Y.M. (email: mori@ sbchem.kyoto-u.ac.jp)

Contents:

Supplementary Figures 1, 2, 3, 4, 5, 6, 7

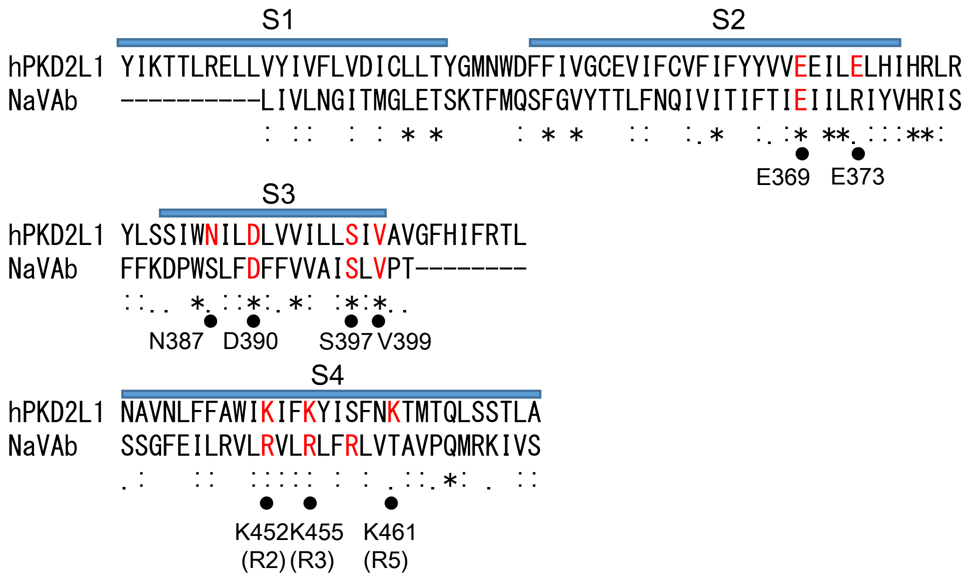


**Supplementary Fig. S1 Comparisons of voltage sensors.** Sequence alignments of the hPKD2L1, and NavAb VS sequences with their corresponding partners. The paring positions in $Na_V$Ab from ref. 48 are annotated by red color in the sequences (see also Supplementary Fig. S3). Secondary structural elements are indicated below the sequence and all critical residues discussed in the text are red colored.

Supplementary Figure 1

PKD2L1 based on PKD2

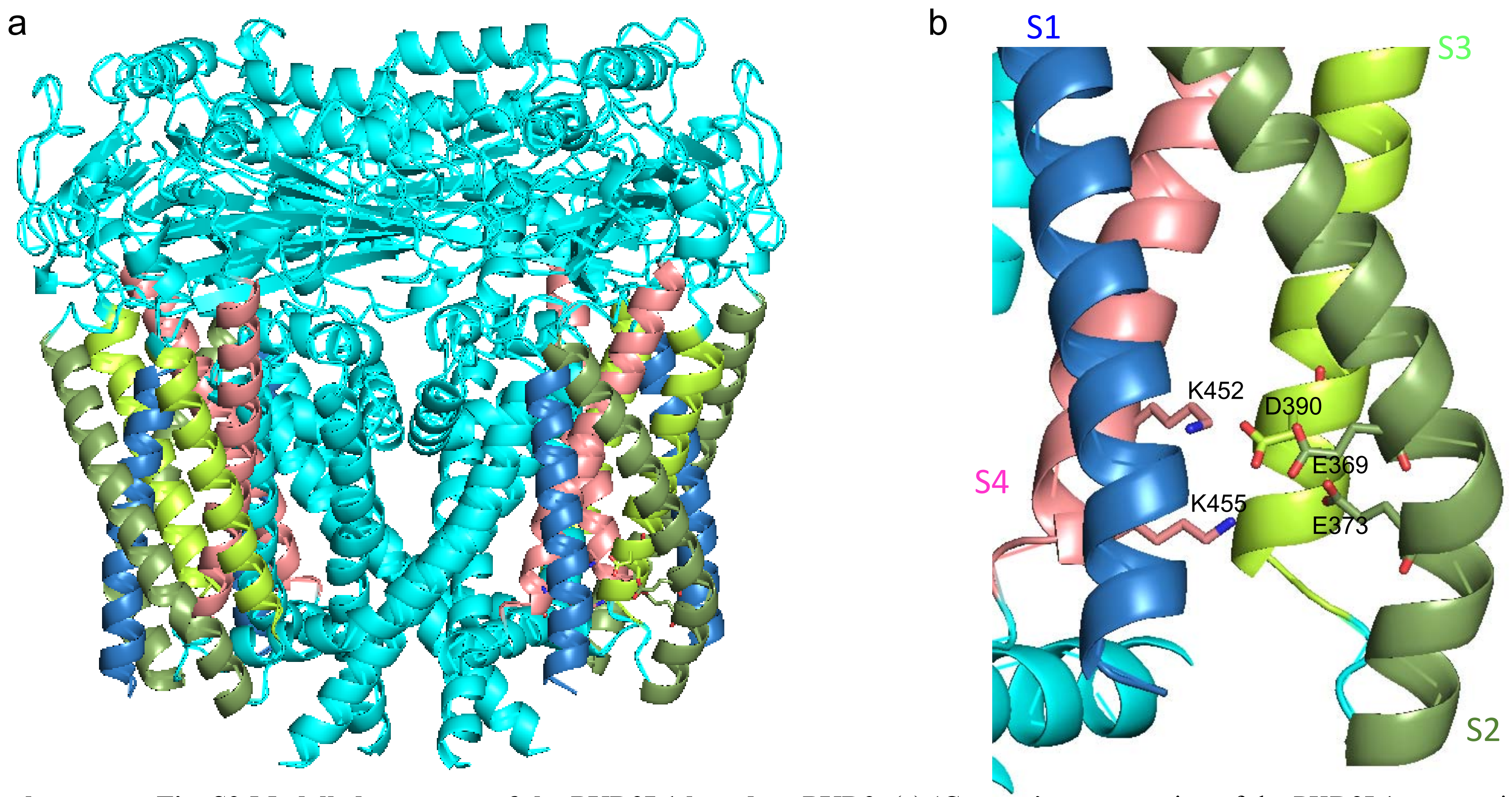


**Supplementary Fig. S2 Modelled structure of the PKD2L1 based on PKD2.** (a) 'Cartoon' representation of the PKD2L1 tetrameric channel. Voltage sensor domain is colored as follows: S1 (blue), S2 (green), S3 (yellow green), and S4 (pink). The other regions are depicted in light blue, for ease of viewing.(b) Structural features that may play a voltage sensor are shown, including a hydrogen bonding interaction that couples the positive charges K452 (PKD2: K405), K455 (K408) and K462 (K415) located in S4, whereas, negative charges paired with positive charged residues in S4 were also conserved E369 (E322) and E373 (E326) in S2, D390 (D343) in S3.

Supplementary Figure 2

PKD2L1 WT based on $Na_VAb$

a b

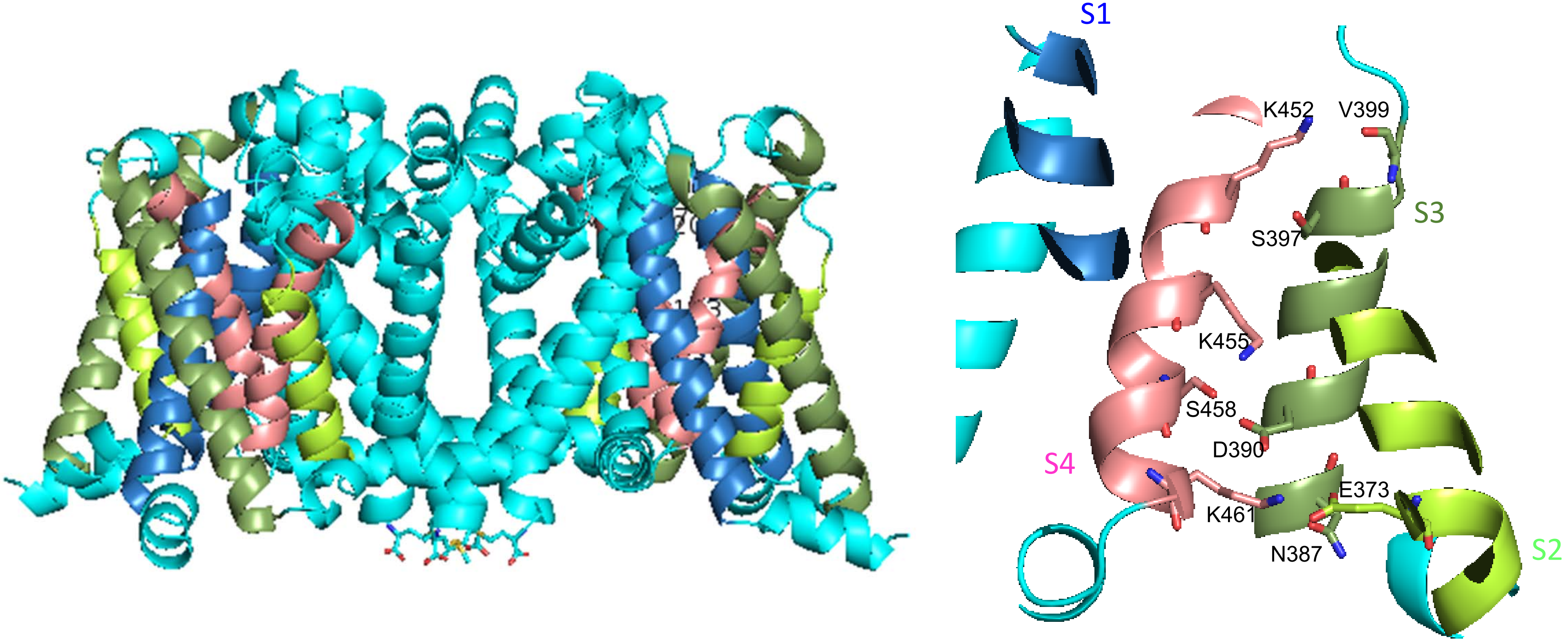


**Supplementary Fig. S3 Modelled structure of the PKD2L1 WT based on $Na_VAb$.** (a) 'Cartoon' representation of the PKD2L1 tetrameric channel. Voltage sensor domain is colored as follows: S1 (blue), S2 (yellow green), S3 (green), and S4 (pink). The other regions are depicted in light blue, for ease of viewing.(b) Structural features that may play a voltage sensor are shown, including a hydrogen bonding interaction that K452 (with V399: 3.44Å), K455 (with S458: 2.81Å) and K461 (with S458: 2.81Å) and K461 (with E373: 2.98Å, D390: 2.97Å, and N387: 2.90Å).

Supplementary Figure 3

PKD2L1 K452Q based on $Na_VAb$

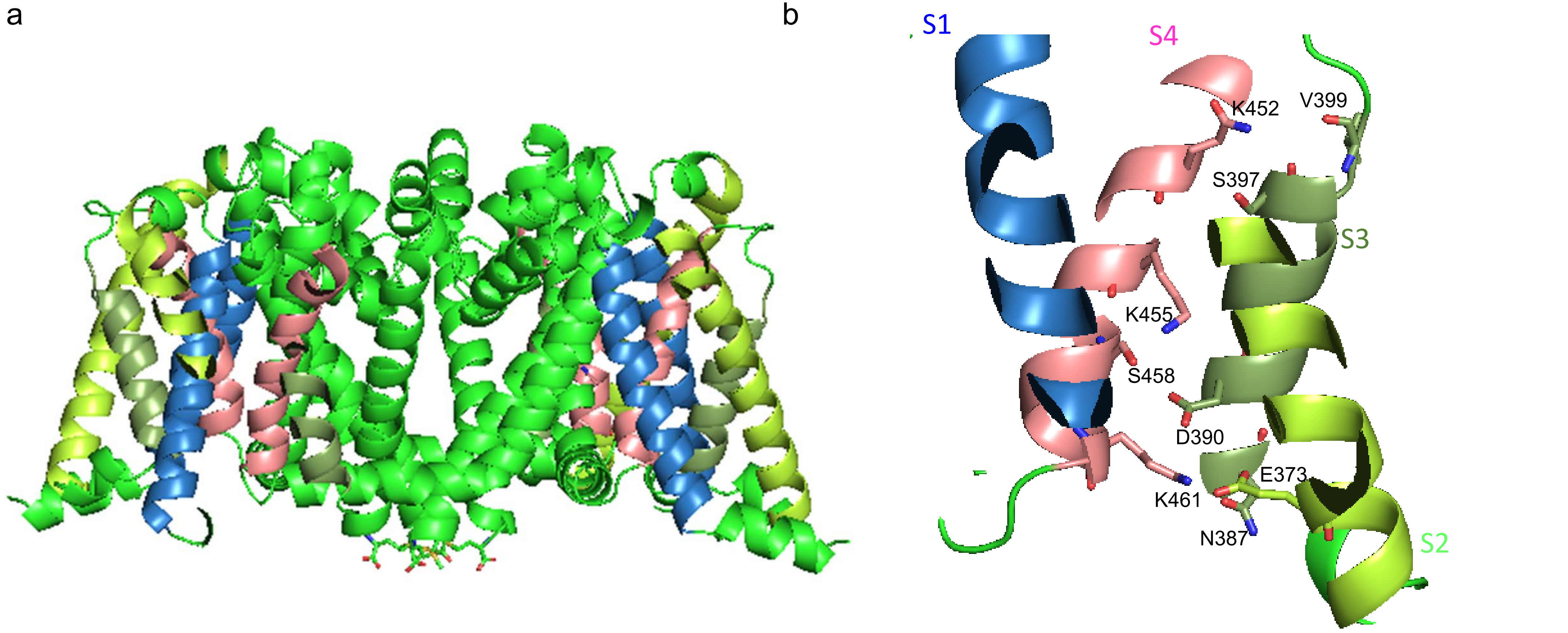


**Supplementary Fig. S4 Modelled structure of the PKD2L1 K452Q based on $Na_VAb$.** (a) 'Cartoon' representation of the PKD2L1 tetrameric channel. Voltage sensor domain is colored as follows: S1 (blue), S2 (yellow green), S3 (green), and S4 (pink). The other regions are depicted in light green, for ease of viewing.(b) Structural features that may play a voltage sensor are shown, including a hydrogen bonding interaction that K455 (with S458: 3.04Å) and K461 (with E373: 2.96Å and with E373: 3.40Å).

Supplementary Figure 4

PKD2L1 WT based on $Na_VAb$

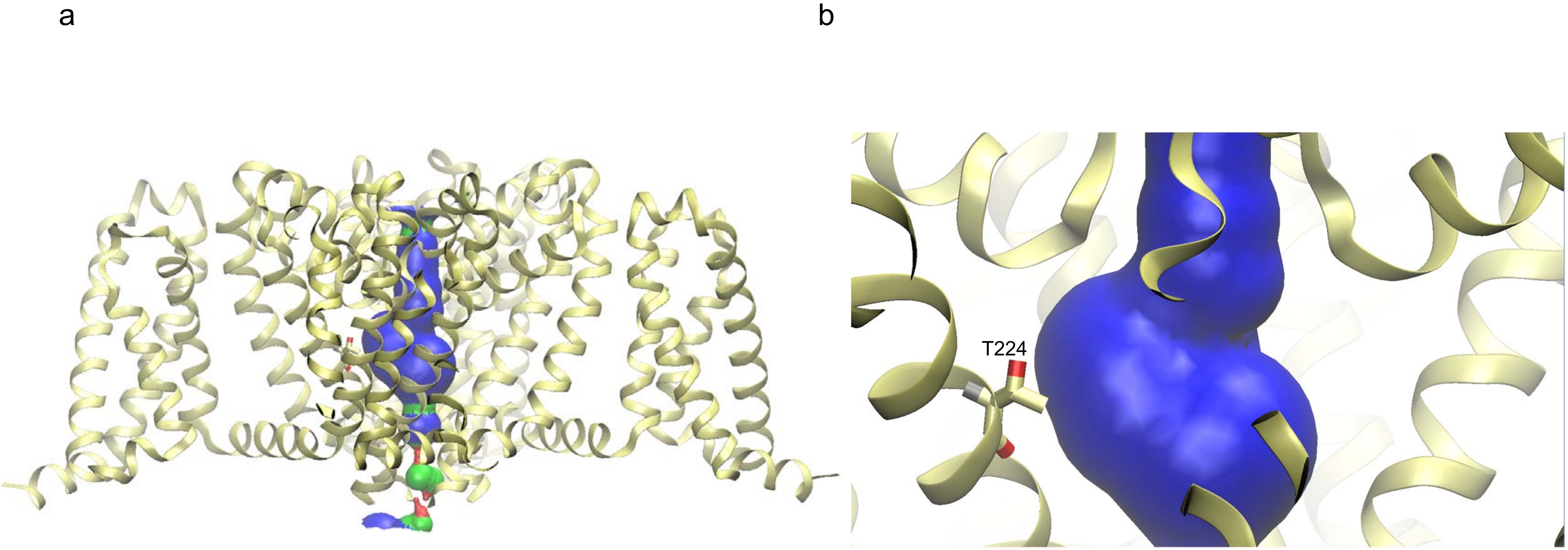


**Supplementary Fig. S5 Simulation of pore sizes.** (a) Surface plots showing the accessible interior of the pore domain calculated with HOLE for PKD2L1 based on $Na_VAb$, where blue surfaces > 2.3Å (maximum pore radius 4.6Å), green surfaces are radii between 1.2 and 2.3Å, and red < 1.2Å. The position of T224 (which corresponds to F502 in PKD2L1) is located at the central cavity near the entrance to selectivity filter.

Supplementary Figure 5

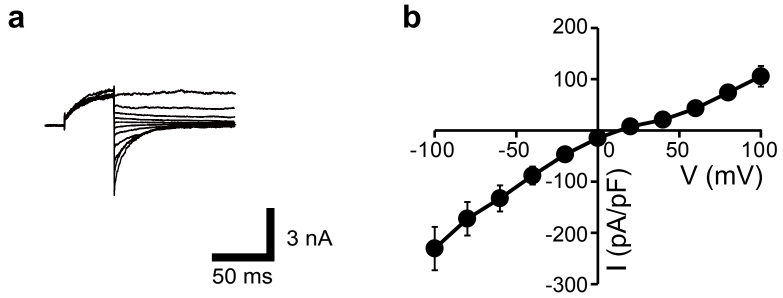


**Supplementary Fig S6. Current-Voltage relationships of PKD2L1 whole cell-current.** **a.** Representative whole-cell currents elicited by step pulses from −100 to +100 mV in 20 mV increments with 40 ms of pre-pulse to +100 mV. **b.** The current–voltage (I-V) relationships for PKD2L1-expressing (filled circles; n=16) HEK293T cells obtained from the traces in a of instantaneous current. Data points are the means ± SEM.

Supplementary Figure 6

a

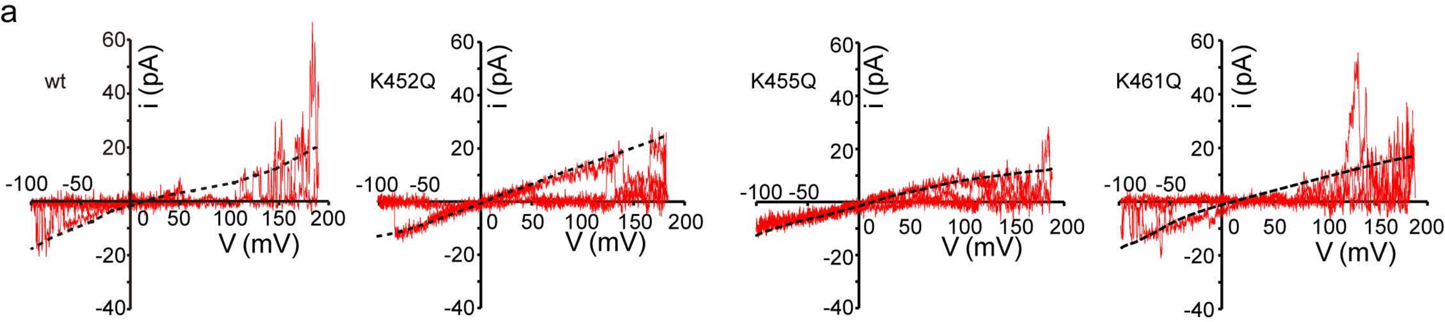


**Supplementary Fig S7. Single channel recordings of PKD2L1 channels under the high-input-resistance whole-cell condition. a.** Representative single channel currents elicited by ramp pulse from −100 mV to +180 mV after reducing the number of available channels by inactivation (holding potential; 0 mV) recorded from PKD2L1-WT (n=15), -K452Q (n=7), -K455Q (n=7), and -K461Q (n=10) expressing HEK293T cells. Each data is composite of three sweeps. Dotted line represent the open channel level.

Supplementary Figure 7
